# Three Nightmare Traits in Leaders

**DOI:** 10.3389/fpsyg.2018.00871

**Published:** 2018-06-04

**Authors:** Reinout E. de Vries

**Affiliations:** ^1^Department of Experimental and Applied Psychology, Vrije Universiteit Amsterdam, Amsterdam, Netherlands; ^2^Department of Educational Science, University of Twente, Enschede, Netherlands

**Keywords:** HEXACO, leadership, personality, contextualized personality, dark triad, STOA, attraction-selection-attrition, career stages

## Abstract

This review offers an integration of dark leadership styles with dark personality traits. The core of dark leadership consists of Three Nightmare Traits (TNT)—leader dishonesty, leader disagreeableness, and leader carelessness—that are conceptualized as contextualized personality traits aligned with respectively (low) honesty-humility, (low) agreeableness, and (low) conscientiousness. It is argued that the TNT, when combined with high extraversion and low emotionality, can have serious (“explosive”) negative consequences for employees and their organizations. A Situation-Trait-Outcome Activation (STOA) model is presented in which a description is offered of situations that are attractive to TNT leaders (situation activation), situations that activate TNT traits (trait activation), and the kinds of outcomes that may result from TNT behaviors (outcome activation). Subsequently, the TNT and STOA models are combined to offer a description of the organizational actions that may strengthen or weaken the TNT during six career stages: attraction, selection, socialization, production, promotion, and attrition. Except for mainly negative consequences of the TNT, possible positive consequences of TNT leadership are also explored, and an outline of a research program is offered that may provide answers to the most pressing questions in dark leadership research.

## Introduction

Interest in leadership traits and the relations between leader personality and leadership styles has waxed and waned over the decades, following the rise and fall in popularity of situational (nurture) and behavioral genetic (nature) explanations of personality and leadership (Judge et al., 2009). Although most researchers nowadays adopt an integrated (“nature in nurture”) stance (e.g., Plomin et al., 2016), models that integrate personality traits, leadership styles, and situations that account for—or can counter—the activation of personality traits and leadership styles, are still rare. This is especially true when considering the dark side of personality and leadership. Although—especially in the wake of several high-profile corporate scandals (e.g., Enron, WorldCom, Volkswagen)—a bourgeoning field of research on dark personality traits (Hogan and Hogan, 1997; Paulhus and Williams, 2002; Chabrol et al., 2009; Buckels et al., 2014) and dark leadership styles (Tepper, 2000; Reed, 2004; Rosenthal and Pittinsky, 2006; Hauge et al., 2007; De Hoogh and Den Hartog, 2008; Schmid Mast et al., 2009; Ghorbani et al., 2010; Schyns and Schilling, 2013; Boddy, 2017; Schmid et al., in press) has emerged, these two fields of research remain by-and-large separate.

As its main contribution the following review offers a theoretical, empirical, and practical integration of personality and (dark) leadership research (1) by proposing that they can—and should—be integrated by conceptualizing leadership styles as *contextualized* personality, (2) by introducing the so-called “*Three Nightmare Traits*” (TNT; de Vries, 2016)—i.e., dishonesty (low honesty-humility), disagreeableness (low agreeableness), and carelessness (low conscientiousness)—as an overarching conceptualization of dark side personality and leadership, (3) by using the *Situation-Trait-Outcome Activation* (STOA) model (de Vries et al., 2016b) as a framework to explain the effects of TNT leaders *on, in*, and *through* situations, and (4) by providing recommendations for organizations how to deal with TNT leaders in different career stages using an extended *Attraction-Selection-Attrition* (ASA; Schneider, 1987) model.

Although most of this review will focus on the TNTs among leaders (hereafter referred to as “TNT leadership”), one of the core assumptions of this review is that leadership styles can be interpreted as contextualized personality traits. That is why, before focusing on the TNT leadership, the following section offers a more general explanation of why there is reason to assume that *all* leadership styles—not only those that are related to the TNT—can be considered contextualized personality traits.

## Leadership styles as contextualized personality

In the following, I will specifically focus on leadership styles. Among leadership scholars, leadership styles—or behavioral tendencies—probably constitute the most common research area. Still, it can be considered a subset of a broader leadership domain, which encompasses, among others, leader knowledge, skills, and abilities [e.g., (emotional) intelligence, leader experience, and leader expertise; (Podsakoff et al., 1983; Cavazotte et al., 2012)], motivation to lead (Chan and Drasgow, 2001), leadership roles (Denison et al., 1995), and leader-subordinate relational quality (Dulebohn et al., 2017) research. Leadership style, as used here, refers to the way a “leader” (i.e., somebody who has gained position power through a process of legitimation) tends to act toward people he or she directs or supervises. Popular leadership styles in the literature include—for example—autocratic and democratic leadership, directive and participative leadership, task- and relation-oriented leadership, charismatic leadership, and transformational and transactional leadership (Bass and Bass, 2009), but next to these mostly “bright” leadership styles, dark leadership styles have received an increasing amount of attention in the last two decades (Schyns and Schilling, 2013).

Contextualization occurs when a relevant context (or frame-of-reference) is added to a (generic or non-contextualized) personality questionnaire (Schmit et al., 1995; Bing et al., 2004). Contextualization can be accomplished by completely rewriting personality items or by using a contextual “tag” to reflect a certain context (e.g., work, home, school, sports, etc…). In the case of leadership, a leadership-contextualized personality questionnaire can be constructed by rewriting personality items to reflect behaviors expressed by somebody in a hierarchical position or to add a tag such as “as a leader” to items. For instance, when contextualizing using a tag, a generic HEXACO (reversed) Agreeableness item “People sometimes tell me that I am too critical of others” (Ashton and Lee, 2009) would become “As a leader, people sometimes tell me that I am too critical of others.”

Contextualized versions of personality scales have been found to be strongly (generally ≥ 0.65) related to their respective generic versions (Bing et al., 2004; Holtrop et al., 2014a,b; Robie et al., 2017) and they generally offer better validities than generic personality scales (Bing et al., 2004; Lievens et al., 2008; Holtrop et al., 2014a; Robie et al., 2017), mainly because contextualized scales reduce within-person inconsistencies in item responding (Lievens et al., 2008). Consequently, leadership-contextualized personality questionnaires are likely to offer better validities in the prediction of leader-relevant outcomes than generic personality questionnaires.

In the following, I will offer five arguments why leadership styles can be considered contextualized personality traits. (1) The *content domain* of leadership styles can be considered a *subset* of personality traits. Whereas personality provides a parsimonious description of *all* possible human behaviors that are psychologically meaningful in *all* possible situations, in line with common definitions of leadership, leadership models restrict themselves to behaviors in a subset of situations, i.e., those that are relevant to the goal-directed (hierarchical) influence of one individual vis-à-vis a group of other individuals. (2) In so far leadership items refer to behavioral tendencies (or: leadership styles) instead of attributions made by subordinates, they are *formulated equivalent* to personality items. Terms that have been used to describe prototypical leadership, such as determined, decisive, organized, responsible, honest, and fair (Lord and Maher, 1993) are the very same terms that have been used in lexical personality studies (Goldberg, 1990; Ashton et al., 2004). Items in leadership questionnaires that describe actual behaviors (e.g., “criticizes poor work;” Fleishman, 1953; de Vries et al., 2002) instead of subordinates' leadership attributions or evaluations, are highly similar to items in personality questionnaires that describe behaviors (e.g., “criticizes others' shortcomings;” Goldberg et al., 2006; see also the HEXACO Agreeableness item above). (3) Empirical evidence shows that leadership styles—like personality traits—are stable across time (Harris and Fleishman, 1955; Dvir and Shamir, 2003; Nielsen et al., 2008; Tafvelin et al., 2011)^1^. (4) Leadership styles show similar levels of *heritability* and genetic correlations show “that there is a *strong common source* [italics added] of genetic variation underlying leadership and personality” (Johnson et al., 2004, p. 31). And, last but not least, (5) there are *strong relations* between personality traits and leadership styles (de Vries, 2012).

Although the first four arguments are theoretically and empirically straightforward, this may not be the case for the last argument. In fact, one of the consistent findings in most studies has been the relatively weak observed relations between personality traits and leadership styles (Judge and Bono, 2000; Bono and Judge, 2004; Lim and Ployhart, 2004; De Hoogh et al., 2005; DeRue et al., 2011), which has led Bono and Judge (2004) to hypothesize that “leadership behaviors are more malleable, more transient, and less trait-like than one might otherwise believe” (p. 906). However, as I've argued elsewhere (de Vries, 2012), the main reason for these relatively weak relations is the fact that all studies included in Bono and Judge's (2004) meta-analysis used leaders' self-ratings of personality and subordinate-ratings of leadership, which introduces an important cross-source upper limit restriction, i.e., that *the maximum possible correlation between two different variables obtained from two different sources is equal to the minimum cross-source correlation of one of these two variables*.

The upper limit of cross-source correlations of the same variable (i.e., self-other agreement) in work settings is generally low; not surpassing the *r* = 0.25 level for leadership (Warr and Bourne, 1999; Ostroff et al., 2004) and *r* = 0.30 for personality (de Vries et al., 2008; Connelly and Ones, 2010)^2^. The fact that none of the meta-analytic zero-order correlations in Bono and Judge's (2004) cross-source meta-analysis surpassed *r* = 0.17 (between extraversion and charismatic leadership), is thus understandable when taking the cross-source upper limit into account. When correcting for low cross-source correlations, de Vries (2012) obtained strong—and consistent—estimates of the relations between personality and leadership styles. That is, charismatic, supportive, and ethical leadership were strongly related to respectively extraversion (β = 0.76), agreeableness (β = 0.74), and honesty-humility (β = 0.50), with only task-oriented leadership having a somewhat weaker relation with conscientiousness (β = 0.33).

These corrected relations offer strong support for a contextualized interpretation of leadership style scales. According to de Vries (2012), charismatic leadership can be considered a contextualized version of extraversion because of the social self-esteem, social boldness, energy, and enthusiasm typical for both extraversion and charismatic leadership; ethical leadership can be considered a contextualized version of honesty-humility because both involve behaviors expressive of sincerity, fairness, and greed avoidance; supportive leadership can be considered a contextualized version of agreeableness (but also some extraversion), because both involve gentleness, patience, flexibility, and tolerance when dealing with subordinates' problems; and finally, task-oriented leadership can be (partly) considered a contextualized version of conscientiousness, because both have to do with order, discipline, and perfectionism when carrying out tasks. Consequently, these four leadership styles—when operationalized as behavioral tendencies—seem to overlap to a large extent with personality traits commonly found in personality models and they may be, accordingly, regarded as contextualized versions of these four traits.

In the following, I will argue that the “negative” pole of three of these four traits are associated with what I will call the “Three Nightmare Traits” (TNT). That is, especially leaders who are characterized by low honesty-humility (henceforth called “leader dishonesty”), low agreeableness (“leader disagreeableness”), and low conscientiousness (“leader carelessness”) may have important negative effects on their subordinates, their organization, and in some cases even society at large.

## Three nightmare traits (TNT) in leadership

To explore the TNTs, it is necessary to first introduce the HEXACO personality model, from which these three traits are derived. The HEXACO model—here applied to leadership—has its basis in lexical personality research. The main assumption of lexical personality research is that anything that can be said about personality is codified in language, and that sufficiently large dictionaries contain a great number of words that may be used to describe somebody's personality (Galton, 1884; Goldberg, 1981). Factor analyses on self- and/or other ratings using these words (most often adjectives) have been applied to uncover the main dimensions of personality. In first instance, lexical personality research (Goldberg, 1990) yielded five main dimensions of personality that are commonly known as the “Big Five.” However, follow-up studies (Ashton et al., 2004; Saucier, 2009; De Raad et al., 2014) have shown that a six-dimensional structure more optimally captures the largest possible cross-culturally replicable personality space in lexical datasets. The dimensions that span this six-factor personality space are commonly known by the HEXACO acronym, i.e., Honesty-humility, Emotionality, eXtraversion, Agreeableness, Conscientiousness, and Openness to experience. Of these six personality dimensions, honesty-humility is least well-captured by the Big Five model, but some of the content associated with emotionality and agreeableness is rearranged in the HEXACO model. The most prominent feature of this rearrangement is that content associated with anger is associated with low HEXACO agreeableness instead of low Big Five emotional stability and content associated with sentimentality is associated with high HEXACO emotionality instead of high Big Five agreeableness (see Ashton et al., 2014, for more details). In this paper, note that when I refer to (leader) dishonesty, disagreeableness, and carelessness, I'm referring to the opposite poles of three of the six HEXACO factors, i.e., low honesty-humility, low agreeableness, and low conscientiousness^3^.

Leader dishonesty, the first of the TNT as applied to leadership, is straightforwardly defined as the opposite pole of HEXACO honesty-humility, i.e., the tendency of somebody (in a leadership position) to be insincere, unfair, greedy, and immodest. Leader dishonesty may be especially problematic for organizations because it may induce, encourage, and/or exacerbate an unethical organizational culture with low trust, low satisfaction, and high turnover. Furthermore, when unchecked it may be associated with serious economic, organizational, and legal costs for an organization. In the popular press, much attention has been devoted to the serious negative effects of dishonest leader behaviors in cases such as the Enron, WorldCom, Volkswagen, and Bernie Madoff scandals, in which CEOs and/or CFOs acted fraudulent and/or condoned fraudulent behaviors. Although there is not much leadership research using HEXACO constructs, HEXACO personality research and leadership research using concepts related to low honesty-humility seem to support the deleterious consequences of leader dishonesty. In personality research, low honesty-humility has been found to be associated with higher levels of counterproductive work behaviors (Zettler and Hilbig, 2010; Wiltshire et al., 2014), workplace delinquency (Lee et al., 2005; de Vries and Van Gelder, 2015), and unethical business decisions (Ashton and Lee, 2008; de Vries et al., 2017). Unethical leadership, which is—when taking into account the self-other agreement problem (see above)—strongly negatively related to honesty-humility (de Vries, 2012), has been found to be related to a more unethical climate or culture (Demirtas and Akdogan, 2015; Eisenbeiss et al., 2015), higher levels of organizational units' deviance/unethical behaviors (Mayer et al., 2009, 2012), lower levels of Organizational Citizenship Behaviors (OCBs; Mayer et al., 2009), lower top team effectiveness (De Hoogh and Den Hartog, 2008), lower levels of trust in the supervisor (Chughtai et al., 2015), lower job satisfaction (Kim and Brymer, 2011; Palanski et al., 2014), lower affective commitment and effort (Brown et al., 2005), and higher intentions to quit (Palanski et al., 2014; Demirtas and Akdogan, 2015). The consequences of leader dishonesty thus seem to be vast, ranging from negative consequences for individual employees and teams to negative consequences for the entire organization's performance (Eisenbeiss et al., 2015).

Leader disagreeableness, the second TNT applied to leadership, is defined as the opposite of HEXACO agreeableness, i.e., the tendency of somebody (in a leadership position) to be unforgiving, overly critical, inflexible, and impatient. Leader disagreeableness may be problematic for organizations because it may induce a culture of fear and retaliation, which may, in turn, lead to high levels of job dissatisfaction, turnover, and costs associated with conflict management and conflict-related lawsuits. It is important to clarify that disagreeableness in the HEXACO model is more closely associated with reactive aggression (vs. reactive cooperation) than with instrumental or proactive aggression (vs. active cooperation). The former is somewhat more closely associated with HEXACO agreeableness, whereas the latter is somewhat more closely associated with honesty-humility (Book et al., 2012; Hilbig et al., 2013; Thielmann et al., 2014; Zhao and Smillie, 2015). Honesty-humility has been found to be more strongly related to premeditated rather than immediate revenge reactions, whereas agreeableness has been found to be fairly equally related to premeditated and immediate revenge reactions following transgressions (Lee and Ashton, 2012). Although it is difficult to extrapolate from Big Five agreeableness because it does not make a clear distinction between active and reactive forms of aggression, thus rendering it unclear whether the following applies to HEXACO agreeableness, teams with lower levels of agreeableness do seem to suffer from lower performance, lower levels of cohesion, more conflicts, and lower levels of workload sharing (Barrick et al., 1998; Peeters et al., 2006; Bell, 2007). In teams, persons with the lowest level of agreeableness seem to have the most negative impact; that is, the least agreeable person in a team has been found to have a greater negative effect on team outcomes than the average agreeableness of a team (Bell, 2007).

In leadership research, HEXACO agreeableness (and not HEXACO honesty-humility) was found to be by far the strongest predictor of leader supportiveness, a measure of the extent to which a leader is considerate toward his/her subordinates, willing to share power, and is non-despotic (de Vries, 2012), and thus leader disagreeableness seems to be associated with low leader support and high leader despotism. Apart from despotic leadership (De Hoogh and Den Hartog, 2008), several other leader constructs exist to measure concepts akin to leader disagreeableness, such as abusive (Tepper, 2000), autocratic/authoritarian (Lewin et al., 1939), destructive (Einarsen et al., 2007), and tyrannical (Hauge et al., 2007) leadership. Despotic leadership (De Hoogh and Den Hartog, 2008) has been found to be negatively related to job performance, OCB, and employee creativity (Naseer et al., 2016). Abusive supervision (Tepper, 2000; Tepper et al., 2017), which has been found to be most strongly related to Big Five agreeableness (Tepper et al., 2001), has been found to be related to higher levels of supervisor-directed, organizational, and interpersonal deviance (Mitchell and Ambrose, 2007; Tepper et al., 2008, 2009), lower levels of perceived interactional or procedural justice and lower levels of employees' OCB (Zellars et al., 2002; Aryee et al., 2007), lower job satisfaction (Tepper, 2000; Tepper et al., 2009), and higher psychological distress and emotional exhaustion (Tepper, 2000; Wu and Hu, 2009). In line with findings on abusive leadership, destructive and tyrannical leadership styles have also been found to be consistently related to negative follower and organizational outcomes (Schyns and Schilling, 2013). A related construct, but with a somewhat different focus, is the construct of autocratic (or: authoritarian) leadership (Lewin et al., 1939). Autocratic leadership, which is defined by unilateral leader decision making and intolerance of disagreement, has been found to result in lower levels of satisfaction (Gastil, 1994), higher levels of cynicism (Jiang et al., 2017), and higher levels of role conflict and role overload (Zhang and Xie, 2017). Probably mostly the intolerance of disagreement inherent in autocratic leadership is associated with higher levels of abusive supervision, making autocratic (i.e., authoritarian) leadership positively related to abusive supervision (*r* = 0.37; Mackey et al., 2017). Boys in Lewin et al. (1939) camp did not perform worse under an autocratic supervisor but reacted more dependent on him and exhibited higher levels of aggression and frustration once the autocratic leader became unavailable (White and Lippitt, 1960).

Note that most of the “abusive” constructs do not separate dishonesty from disagreeableness, and thus most—if not all—are probably related to both leader dishonesty and leader disagreeableness. For instance, abusive leadership was found to be almost equally negatively related to HEXACO honesty-humility and agreeableness (Breevaart and de Vries, 2017), and thus it may be unclear, when investigating its effects, which consequences are due to leader dishonesty and which are due to leader disagreeableness.

Leader carelessness, the third of the TNT traits applied to leadership, is defined as the opposite of HEXACO conscientiousness, i.e., the tendency of somebody (in a leadership position) to be sloppy, lazy, negligent, and impulsive. Leader carelessness may be problematic for organizations, because it may be associated with an accident-prone culture, in which rules and regulations are disregarded and in which industry standards, necessary for optimal performance, are violated. More generally, it may lead to a culture in which low, instead of high, performance is the norm. When related to leadership, conscientiousness as a personality variable has been found to be most closely associated with task-oriented or structuring leadership (de Vries, 2012; Babiak et al., 2017), although relations with ethical leadership and leader consideration have also been noted (DeRue et al., 2011; Babalola et al., in press). One of the most notable characteristics of “careless” people with low levels of conscientiousness is their enhanced level of procrastination, i.e., their tendency to delay tasks that need to be done. In a meta-analysis by Steel (2007), procrastination was very strongly negatively related (*r* = −0.62) to conscientiousness. Another characteristic of carelessness is low levels of self-control. Of all personality traits, conscientiousness has been found to be by far the strongest correlate of self-control (e.g., *r*'s > 0.50; de Vries and Van Gelder, 2013). A third characteristic of careless people is that they are more likely to make errors and to be involved in accidents because they are less motivated to follow safety regulations (Wallace and Vodanovich, 2003; Clarke and Robertson, 2005; Christian et al., 2009). Consequently, careless leaders are more likely to put things off until tomorrow which should be done today, they are more likely to lack a sense of urgency and discipline, they are more likely to make errors or let errors go unnoticed, and they are more likely to seek out pleasurable activities instead. Such a profile of low self-control, high procrastination, and high error proneness is probably best reflected in laissez-faire leadership. Meta-analyses seem to confirm a negative relation between conscientiousness and laisser-faire leadership (Bono and Judge, 2004; DeRue et al., 2011). In turn, task-oriented leadership and laissez-faire leadership have been found to be important predictors of outcome variables. That is, low task-oriented leadership has been associated with low levels of leader effectiveness (but not lower levels of job and leader satisfaction) and high levels of laissez-faire leadership has been associated with both low levels of leader effectiveness and low levels of job and leader satisfaction (DeRue et al., 2011).

One might question whether passive leadership such as laissez-faire leadership and lack of task-oriented leadership constitute such a liability to the organization to call leader carelessness a “nightmare trait.” As Einarsen et al. (2007) argue, the answer should be an unequivocal “yes,” because passive leadership not only constitutes shirking functional responsibilities, which can thus be considered stealing company time, but because it may also result in highly negative consequences for organizations when crucial errors are made or when important safety regulations are violated. Given the fact that passive leadership (cf. leader carelessness) has been strongly negatively associated with positive organizational outcomes (DeRue et al., 2011), it may be appropriate to label it—following Einarsen et al. (2007)—as a destructive leadership style. Although too high levels of conscientiousness may be (but only slightly) “too much of a good thing” (Le et al., 2011), and too high levels of leader perfectionism may result in negative consequences associated with micromanagement, too high levels of leader carelessness seem to result in much worse outcomes in terms of decreased individual, team, and organizational effectiveness.

## Combining the TNT with extraversion, emotionality, and openness to experience

The three remaining HEXACO dimensions, extraversion, emotionality, and openness to experience, do not seem to be associated to the same degree with negative leadership outcomes as the TNTs (but see Judge et al., 2009 for possible negative leadership outcomes associated with either low or high extraversion, emotional stability, and openness to experience). However, in some instances, combinations of the three remaining traits with the TNT may be associated with even worse outcomes. The most important of the remaining traits is extraversion. Extraversion is one of the most robust correlates of leader emergence, transformational/charismatic leadership, and leadership effectiveness (Judge et al., 2002; Bono and Judge, 2004; de Vries, 2012). However, in combination with leader dishonesty, leader disagreeableness, and leader carelessness, an extravert leader may turn out to be even more destructive, showing characteristics of what has been called a personalized (i.e., self-aggrandizing, non-egalitarian, and exploitative) charismatic leader (McClelland, 1975; House and Howell, 1992), who misuses his/her charisma and dominance to obtain personal goals at the expense of others. Interestingly, House and Howell (1992) described in detail the pattern of personalized charisma using narcissism, Machiavellianism, and authoritarianism—traits that are associated with leader dishonesty and leader disagreeableness. Together with psychopathy, narcissism and Machiavellianism form the so-called dark triad, which are associated with grandiosity, entitlement, and feelings of superiority (narcissism), manipulativeness and deception (Machiavellianism), and antisocial tendencies, glibness, lack of empathy, and irresponsibility (psychopathy). Recently, a fourth trait, sadism, has been added to the dark triad to form the dark tetrad (Chabrol et al., 2009; Buckels et al., 2014), the core of which is formed by the enjoyment of physical and/or emotional pain in innocent others through aggressive and/or cruel acts.

Although the dark triad (and tetrad) have been found to be related to especially low agreeableness in the Five-Factor Model (FFM; O'Boyle et al., 2015), the most important correlate of the dark triad (and tetrad) is HEXACO honesty-humility. Through the inclusion of honesty-humility, the HEXACO model has been able to outperform the Big Five model (or: FFM) in the explanation of not only the dark triad (Lee and Ashton, 2005, 2014), but also the dark tetrad (Book et al., 2016). Although the common core of the dark triad/tetrad traits, which are generally strongly related to each other, is formed by honesty-humility, each of the dark traits have some residual relations with other HEXACO traits. That is, besides honesty-humility, narcissism has also been found to be positively related to extraversion, Machiavellianism negatively to agreeableness, and psychopathy negatively to emotionality and to conscientiousness (Lee et al., 2013). Sadism has been found to be most closely related to low honesty-humility and low emotionality, but also (but less strongly) low agreeableness and low conscientiousness (Book et al., 2016).

The core of these dark triad/tetrad traits thus seems to be formed especially by low honesty-humility (i.e., dishonesty), but also somewhat low agreeableness (i.e., disagreeableness) and low conscientiousness (i.e., carelessness). A profile that combines high levels of extraversion with leader dishonesty is indicative of leader narcissism whereas a profile that combines low levels of emotionality with the TNT (i.e., leader dishonesty, leader disagreeableness, and leader carelessness) is indicative of psychopathic leadership. Consequently, the most “dangerous” leaders seem to be those leaders who combine the TNT traits with high extraversion and low emotionality, resulting in a narcissistic-psychopathic leadership profile.

It is somewhat less clear what the results may be of a leader profile, which combines the TNT with low or high openness to experience. Openness to experience, like extraversion, has been found to be positively related to leader emergence, leader charisma, and leader effectiveness (Judge et al., 2002; Bono and Judge, 2004), and thus it may be true that, just like extraversion, high openness to experience strengthens the negative effects of the TNT on individual, team, and organizational outcomes. On the other hand, high openness to experience, when expressed through new ideas and methods, may also distract or even compensate for some of the negative effects associated with the TNT.

Although there is, at present, not much evidence on profiles that combine the TNT with the other three personality traits, some studies suggest that outcomes may be worst when combining low honesty-humility with extraversion. For instance, Gylfason et al. (2016) found that respondents high on extraversion and low on honesty-humility were most likely to send deceiving messages in a “cheap talk” game. Similarly, in two of the three samples investigated, Oh et al. (2011) found that extraversion and honesty-humility interacted in the prediction of workplace deviance, such that the highest level of workplace deviance was observed for those high on extraversion and low on honesty-humility. Furthermore, narcissistic leadership, a leadership style which combines high extraversion with low honesty-humility, has been found to be associated with problematic organizational and/or societal outcomes, such as higher levels of tax evasion (Olsen and Stekelberg, 2016), higher numbers of lawsuits (O'Reilly et al., 2018), higher levels of actual fraud (Rijsenbilt and Commandeur, 2013), and more volatile and extreme (both negative and positive) return on assets (Chatterjee and Hambrick, 2007).

With respect to psychopathic leaders (i.e., those leaders who combine the TNT with low emotionality), Babiak et al. (2010) found—based on observational ratings—that 5.9% of their sample consisting of managers and executive had a “potential psychopathy” score. Although, based on 360° ratings, these managers were perceived to have good communication skills and innovative ideas (indicative of respectively high extraversion and high openness to experience), psychopathy scores correlated negatively (*r* = −0.41) with supervisory performance ratings. That is, although people with psychopathic profiles were able to successfully climb the corporate ladder, probably due to their high extraversion and high openness to experience, they were found to have a negative impact on the team and the organization when considering their performance evaluations.

## The STOA model of TNT leadership

Whether and how people emerge as leaders, act as leaders, and are effective as leaders, can only be ascertained by taking situational contexts into account. People act *on, in*, and *through* situations, and thus any model that describes leadership needs to also describe how the personality of leaders “unfolds,” i.e., what situations (potential) leaders seek out, in what way they behave in these situations, and what the effects are of their behaviors. The STOA model posits three activation mechanisms that describe the way personality unfolds: (1) a situation activation mechanism, (2) a trait activation mechanism, and (3) an outcome activation mechanism (de Vries et al., 2016b). First of all, based on their personality, people perceive, select, manipulate, and/or evoke situations to “fit” their personality (Buss, 1987, 2009). To become a leader, persons have to first of all select situations that afford them to become a leader. People who avoid social settings, because they feel less comfortable in groups or because they are less interested in social situation, are unlikely to become leaders in the first place. People low in extraversion and high in emotionality/anxiety are not only less interested in social situations (Holtrop et al., 2015), with extreme levels of these traits they may also be more likely to actively avoid such situations because of social phobia (Kotov et al., 2010). Highly extraverted people, in contrast, seek out social situations, not only because such situations are rewarding or because they like social occasions, but especially because they seek social attention (Ashton et al., 2002). Thus, by virtue of their personality, extraverted people are more likely to seek out situations in which they can fulfill a leadership role.

Social situations, in turn, afford the expression of leadership-related traits. Trait activation, the second of the proposed mechanisms, is predicated on trait activation theory (TAT; Tett and Burnett, 2003), which maintains that traits only get activated when situations allow these traits to be expressed. Social situations may activate several traits, but for leadership, especially three personality dimensions seem to be most relevant: extraversion, conscientiousness, and openness to experience. These are the three personality dimensions that have been found to be most strongly and positively related to leader emergence (Judge et al., 2002; Ilies et al., 2004; Reichard et al., 2011). These are also the three personality dimensions that have been found to be most strongly related to proactive personality (a.k.a. proactivity, see de Vries et al., 2016c), which includes taking charge, networking, voice behaviors, and career initiative; behaviors that can only be expressed in social situations and that are viewed as indicating leader potential (Fuller and Marler, 2009). With respect to extraversion, especially social boldness may play a role. People who are socially bold are more likely to take charge in groups. With respect to conscientiousness, especially diligence and organization may play a role. People who are diligent and organized, work hard and plan carefully in order to have a better chance to reach their goals; traits that also seem to help groups to become successful (Peeters et al., 2006; Bell, 2007). With respect to openness to experience, especially creativity, and innovativeness may play a role, behaviors that may help groups distinguish themselves through new and original solutions. People who have such a profile of high extraversion, high conscientiousness, and high openness to experience are likely to be viewed as an important asset to a group (i.e., obtain “idiosyncrasy credits;” Hollander, 1992), and are consequently more likely to emerge as a leader.

Two other traits that have been proposed to be relevant to leader emergence and that may be activated in social situations are narcissism and self-monitoring. As noted above, narcissism has been found to be related to both (low) honesty-humility and (high) extraversion (Lee et al., 2013). Several studies have argued that narcissism is related to leader emergence (Paunonen et al., 2006; Nevicka et al., 2011), even when correcting for Big Five extraversion (Brunell et al., 2008), suggesting that low honesty-humility (especially low modesty) may play a role. Similar to narcissism, self-monitoring has also been found to be related to (low) honesty-humility and (high) extraversion (Ogunfowora et al., 2013), and also similar to narcissism, self-monitoring has been found to be positively related to leader emergence (Ellis, 1988; Zaccaro et al., 1991). Furthermore, Foti and Hauenstein (2007) found that a pattern that combined high levels of (social) dominance (which has been conceptualized as a facet of extraversion; e.g., Lee and Ashton, 2004), intelligence, self-efficacy, and self-monitoring had the strongest correlation with peer and superior ratings of leadership impressions. However, a recent meta-analysis on the relation between narcissism and leader emergence found that, when correcting for extraversion, the positive relation between narcissism and leader emergence turned to near zero (Grijalva et al., 2015). Because self-monitoring relates to extraversion as well, it looks as though variance associated with extraversion is the only real and substantial correlate of leader emergence in these two traits.

Outcome activation, the third of the proposed mechanisms, pertains to the effects that activated traits have. Three kinds of effects may be distinguished: (1) recognition, (2) perception, and (3) attribution. In the first place, one of the main outcomes of socially bold, disciplined/organized, and/or creative behaviors is that group members take notice. That is, people only get “recognized” as a potential leader if they show prototypical leader behaviors. Second, the more a person acts socially bold, disciplined/organized, and/or creative, the higher the chance that group members act upon that person's suggestions, which strengthen leadership perceptions. And third, if—by following the suggestions of somebody who shows prototypical leader behaviors—a group becomes successful, the results are likely to be attributed to the person who has shown leaderlike behaviors, resulting in even stronger leadership perceptions (cf. the Romance of Leadership theory, Meindl, 1995; see also de Vries, 2000). In general, holding everything else constant, socially bold, disciplined, and creative behaviors (i.e., proactivity) are more likely to result in positive outcomes for a group than behaviors that are their opposites (i.e., socially phobic, unorganized, and uncreative). That is, proactive personality has been shown to be one of the most important predictors of job performance and business success (Rauch and Frese, 2007; Fuller and Marler, 2009; Thomas et al., 2010).

Apart from conscientiousness, the main drivers of leader emergence thus appear to be traits that are *not* aligned with the TNT. However, apart maybe from carelessness (i.e., low conscientiousness) which may be associated with higher number of mistakes Wallace and Vodanovich, 2003; Clarke and Robertson, 2005; Christian et al., 2009, there does not seem to be anything in the two remaining TNT traits, i.e., dishonesty and disagreeableness, that prevents people who exhibit these traits to rise through the ranks and to obtain a leadership position. Elsewhere (de Vries, 2016; de Vries et al., 2016b), it has been argued that some situations are sought out by people who are characterized by higher levels of dishonesty, disagreeableness, and carelessness because these types of situations allow people to more readily express these traits, free from constraints. That is, people high on dishonesty are more likely to seek out situations that *allow for exploitation* (Sherman et al., 2015), because in such situations they can more readily express dishonest behaviors (Hilbig and Zettler, 2009; Hilbig et al., 2012) and because in such situations, they are more likely to obtain “sex, power, and money” (Lee et al., 2013). People high on disagreeableness are more likely to pay attention to negative events (Bresin and Robinson, 2015) and seek out situations that *allow for (interpersonal) obstruction* (Rauthmann, 2012; de Vries et al., 2016b), and are consequently more likely to have relationship conflicts (Bono et al., 2002). Disagreeableness may result in positive outcomes for a person if s/he has enough power and status to get more easily what s/he wants using disagreeable behaviors (Sell et al., 2009). Last of all, people high on carelessness are more likely to seek out situations in which they can *shirk duties* and avoid planning and goal-setting, because especially in situations in which they have to set goals and perform (e.g., in most school and organizational settings), carelessness (i.e., low conscientiousness) is associated with lower performance (Barrick and Mount, 1991; Poropat, 2009).

In fact, some studies suggest that norm violating behaviors (i.e., dishonest, disagreeable, and/or careless behaviors) may be perceived as leaderlike, because they suggest to others that the norm violator has the power to act free from social constraints (Van Kleef et al., 2011). Power derived from a leadership position, in turn, may free people to “do as they please” (Galinsky et al., 2008), resulting in a greater likelihood to express norm violating behaviors. That is, individuals high on the TNT are more likely to seek out situations in which they can freely express counternormative traits (situation activation). Combined with high levels of extraversion (social boldness) and openness to experience (creativity), the expression of TNT behaviors may be perceived as more leaderlike, which may make it more likely for them to emerge as a leader. In turn, when they have power, TNT leaders may feel less constrained, resulting in more frequent and open expression of the TNT (trait activation), which may result in positive outcomes for the self in terms of “sex, power, and money” (outcome activation; Lee et al., 2013), especially when there are no countervailing powers (i.e., checks and balances). Evidence of the STOA mechanisms is, for instance, found in a study by Wisse and Sleebos (2016), who observed a positive relation between supervisor-rated Machiavellianism and his/her perceived position power (indicative of both situation activation and outcome activation) and who found that Machiavellianism interacted with perceived position power in the prediction of subordinate-rated abusive supervision. That is, abusive supervision of Machiavellian leaders was higher when the supervisor had more position power. Together with the finding that Machiavellianism is positively related to career success in terms of a (higher) leadership position (Spurk et al., 2016), the results seem to suggest that norm violation may indeed be beneficial for perpetrators.

## Nightmare careers

What should organizations do when faced with a TNT leader? And are there ways to prevent TNT leaders to rise through the ranks? In the following, I'll use an extended version of the ASA model of Schneider (1987), including six (instead of Schneider's three) career phases, i.e., attraction, selection, socialization, production, promotion, and attrition, to describe possible actions organizations can take to prevent TNT applicants for leadership positions to become—in the end—TNT CEOs. Following de Vries (2016), attraction is the phase in which recruitment efforts take place, selection the phase in which a candidate is chosen from the available applicant pool, socialization the phase in which a new leader formally and informally gets to know his/her team and organization, production the phase in which a leader performs in his/her job, promotion the phase in which a leader qualifies for an even higher-level position, and attrition the phase in which a contract is (voluntarily or involuntarily) terminated. In Table 1, an overview is offered of the TNTs, in what situations these traits are activated, what possible negative outcomes are associated with the TNTs, and what organizations can do to prevent situation, trait, and outcome activation of these traits among leaders^4^.

**Table 1:** Implications of the TNT for attraction, selection, socialization, production, promotion, and attrition in organizations.

	**Dishonesty**	**Disagreeableness**	**Carelessness**
*Behaviors*	Insincere, Unfair, Greedy, Immodest, Manipulative	Unforgiving, Aggressive, Intolerant, Stubborn, Inflexible	Unorganized, Lazy, Sloppy, Impulsive, Procrastinating
*Situation Activation*	Dishonest leaders seek out situations that afford **exploitation**	Disagreeable leaders do not shy away from situations that afford **obstruction**	Careless leaders avoid situations which afford **duty** and seek out situations that afford impulse gratification
*Trait Activation*	Situations that afford exploitation activate dishonest behaviors	Situations that afford obstruction activate disagreeable behaviors	Situations that afford duty activate conscientiousness vs. carelessness
*Outcome Activation*	**Personal benefits**: status, power, money **Organizational costs**: distrust, dissatisfaction, and turnover; organizational, economic, and legal costs	**Personal benefits**: power due to conformism and fear employees **Organizational costs**: culture of fear, conflicts, dissatisfaction, employee turnover, lack of checks and balances	**Personal benefits**: low energy costs when relying on work of others **Organizational costs**: reactive management, planning problems, errors, low performance, dissatisfied clients
*Attraction*	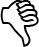 : Advertise high salary and bonuses, quick promotion procedures, fast sector growth, and high company status 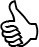 : Advertise the importance of ethical leadership and societal (instead of personal) relevance of work	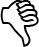 : Advertise ruthless corporate atmosphere, cutthroat competition, “do or die” leader mentality 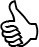 : Advertise the importance of leader support, compromise, acceptance of others' opinions, tolerance of diversity, and intolerance of bullying	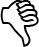 : Advertise fringe benefits such as time off from work and business trips 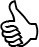 : Advertise the importance of managerial competencies, complete planning, specific goal-setting, being organized, showing self-discipline, and being perfectionistic
*Selection*	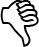 : Failure to include an integrity survey and/or ethical dilemmas in the interview, and failure to include reference and cv-checks 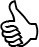 : Inclusion of reliable and valid integrity instruments and checks in the entire selection	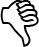 : “Toughness” evaluated in terms of positive leadership qualities; failure to check for interpersonal conflicts at previous employer 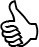 : Check reactions to employee mistakes (forgiveness and use of mistakes for learning); check previous employer on handling of conflicts	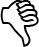 : Neglect sloppy cv, unstructured writing, and spelling mistakes; failure to check leader performance indicators in previous job 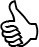 : Evaluate tidiness cv; use work sample tests to check managerial planning/ goal-setting competencies; check leader performance indicators and work outcomes previous job
*Socialization*	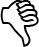 : Start out by explaining status hierarchy at work; show admiration for status, power, and money; provide examples of shady practices that helped the organization 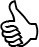 : Ethics training and open discussion of ethical dilemmas; equal treatment of top and work floor (approachable CEO)	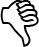 : Focus on negative behaviors that “deserve” punishment; providing negative example behaviors of intolerance to mistakes, personal criticism, and lack of forgiveness 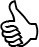 : Provide positive example leader behaviors focusing on learning from mistakes, adequately dealing with gossip, and respectful conflict resolution	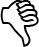 : Focus on “fun” instead of on work-related issues; showing an “anything goes” mentality with respect to tasks, deadlines, time at work, and work-related goals 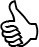 : Discuss and promote healthy work-home balance and balance between discipline and fun at work; promote healthy planning and perfectionism, and promote learning from mistakes
*Production*	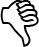 : No ethical guidelines, no clear responsibilities at work; no in- and output control systems; interpreting norm violating behaviors in terms of leadership 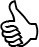 : Having an ethical and transparent culture; checks and balances on use of power, safeguards (multiple eyes) for moral dilemmas	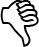 : Failure to quickly act on conflict behaviors, aggression, and bullying; failure to define positive alternatives and consequences of misbehaviors 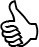 : Having a confidential counselor for victims of bullying and intimidation; having leaders learn how to adequately intervene and deal with conflict situations, anger, and intimidation	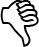 : No in- and output control systems, no planning, feedback, and goals, no consequences for sloppy and/or late work 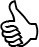 : Top management shows an interest in work (in- and output) and provides specific feedback on plans, goals, and on content of work; a culture that supports learning from mistakes, a healthy work-home balance, punctuality, and perfectionism
*Promotion*	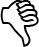 : Interpreting low humility and acts of Machiavellianism as a sign of leadership 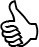 : Promotion based on self-sacrifice, OCB, lack of status orientation, and real signs of humility; coaching, supporting, and stimulating humble employees who decline promotion offers	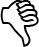 : Promotion based on “law of the jungle;” supporting or even encouraging acts of aggression to reach the top 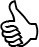 : Promotion based on ability to support others and to resolve conflicts without resorting to intimidation tactics, and to help others learn from their mistakes—i.e., authority instead of authoritarianism	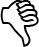 : Promotion not based on task competencies and personal accomplishments but on looking busy; interpreting having others do the tasks as a sign of leadership 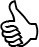 : Promotion based on thorough evaluation of leader task performance, task competencies/expertise, and top management leadership potential
*Attrition*	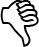 : No records of unethical leadership behaviors; receptiveness top management for manipulations and charm 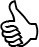 : Adequate records on (un-)ethical leadership; top management receives feedback from all levels in the organization	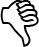 : No records of conflicts and bullying; top management lack ties with vulnerable employees in the organization 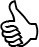 : Adequate records on supportive leadership behaviors; top management relates to vulnerable employees and can adequately judge escalating (or de-escalating) behaviors	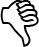 : No managerial performance records; no record on whether somebody makes plans, sticks to them, reaches his/her goals, or shirks his/her duties 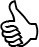 : Adequate records on task-oriented leadership, regular performance appraisals using clear and objective indicators of somebody's managerial competencies/performance

*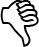/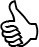: Actions of the organization that may strengthen/weaken nightmare traits*.

### Attraction

To attract employees for leadership positions, firms are likely to use a great number of recruitment channels to find motivated candidates (Russo et al., 2000). From the perspective of the recipients of the recruitment messages, these messages may either generate interest in the organization or not. In terms of the STOA model, *situation activation* is the main mechanism in the attraction phase. Prospective employees are mainly attracted to organizations based on the perception of the nature of work and the organizational culture (Boswell et al., 2003; Chapman et al., 2005). Whereas vocational interests are the most important determinant of vocational (job) choice (Tracey and Hopkins, 2001; Volodina and Nagy, 2016), which plays a role in the earlier phase of a career, personality may play an important role in determining organizational culture preference in later career stages. Only few studies have been conducted on the relations between personality and organizational culture preference, and none have been conducted using the HEXACO model, but findings do suggest that personality plays an important role in line with the TNT described above. That is, of all relations explored between self- and peer-reported personality and self-reported organizational culture preference, Judge and Cable (1997) found agreeableness to be the most important negative predictor of an aggressive organizational culture preference, suggesting that people with a high level of TNT disagreeableness are more likely to apply for an organization which is more likely to condone aggression. The second most important relation was between conscientiousness and preference for an outcome-oriented culture, suggesting that careless people are more likely to apply for an organization that is less outcome-oriented. In a sample of students, attractiveness of a sales job with “out of town travel” was highest among students with low conscientiousness and low agreeableness (Stevens and Macintosh, 2003), suggesting that careless and disagreeable people are more likely to apply for organizations that offer these types of “away-from-work” fringe benefits. With respect to dishonesty, low scorers on honesty-humility are motivated by wealth, privilege, and status (Lee and Ashton, 2004), so it may seem logical to assume that organizations that “flaunt” these kinds of characteristics, are more likely to be attractive to dishonest people. Empirical evidence suggests that this is indeed the case; i.e., people low on honesty-humility are more likely to be attracted to power and money than people high on honesty-humility (Lee et al., 2013). Furthermore, Ogunfowora (2014) found that people low on honesty-humility, but not people high on honesty-humility, were more likely to be attracted to an organization with a CEO who was morally questionable.

### Selection

From an organizational perspective, *trait activation* is the most important mechanism in the personnel selection phase. In this phase, organizations provide candidates with situations (e.g., questions in interviews and selection assessments) that activate traits and skills that are deemed relevant by the organization. With respect to the TNT, there is convincing evidence that especially carelessness (i.e., low conscientiousness), but also dishonesty (low honesty-humility) are associated with higher counterproductive behaviors and lower job performance (e.g., Barrick and Mount, 1991; Ones et al., 1993, 2007; Ones and Viswesvaran, 1998; Schmidt and Hunter, 1998; Sutin et al., 2009; Fine et al., 2010; Brown et al., 2011). Although there is no evidence for the negative effects of disagreeableness from personnel selection studies, team studies seem to suggest that one disagreeable team member can have a strong negative effect on team cohesion (Barrick et al., 1998; Peeters et al., 2006; Bell, 2007; O'Neill and Kline, 2008), which may be exacerbated when the team leader is disagreeable. Apart from the TNT, extraversion has been found to be related to career success and leadership position, in part because of its relation with perceptions of charisma (Vergauwe et al., 2017). As argued above, although organizations might like to select on extraversion to recruit potential leaders, especially the combination of extraversion with the TNT may have negative consequences for an organization. Both narcissism (indicative of leader dishonesty and extraversion) and Machiavellianism have been found to be associated with positive career outcomes for the employee him-/herself, such as higher salary (narcissism) and higher leadership position (Machiavellianism) (Spurk et al., 2016), but mostly negative outcomes for the organization (Spain et al., 2014). Consequently, doing a thorough background check and making sure that the selection procedure allows the measurable expression of the TNT through (reliable and valid) structured interviews, questionnaires, or assessment tools, seems to be important to select non-TNT leadership candidates and, consequently, to prevent potential toxic organizational consequences.

### Socialization

In the socialization (or: onboarding) phase, new employees (including those who applied for a leadership position) get to evaluate the actual level of *trait activation* and *outcome activation* that the job and the organization offer. This phase is important for the establishment of a psychological contract (Kotter, 1973), an informal set of reciprocal expectations between an employee and his/her organization. These expectations cover the kind of behaviors that are allowed and/or expected at work and the kind of outcomes expected of an employee. Based on these informally and/or formally communicated expectations, new employees/leaders learn whether the organization affords or constrains TNT-based behaviors and what outcomes result from such expressions of the TNT. An example of an onboarding activity is ethics training. Although the effect of limited ethics training has been found to be transient (Richards, 1999), more exhaustive and in-depth ethics training has been found to have a longer lasting effect on ethical decision-making (Mumford et al., 2008) and to have a positive effect on the perceived ethical culture of an organization (Valentine and Fleischman, 2004). Although it is unlikely that ethics training changes a person's personality, it does make an employee aware of the norms and values of an organization, which may limit the expression of nightmare traits (i.e., prevents trait activation) and which may limit expectations that positive outcomes may result from the expression of nightmare traits (i.e., prevents outcome activation). Because the socialization phase for leadership positions is often short and new leaders are often expected to make changes to their team and/or organization, a potential danger is that ethics training or attention to ethical dilemmas have limited effect and that the first thing TNT leaders do is to try to make their mark by changing the culture of the organization to fit their personality.

### Production

All three STOA mechanisms play a role in this phase. That is, a new leader is likely to try to seek out certain organizational situations and/or to change them to fit his/her personality (*situation activation*), these situations are likely to activate (combinations of) his/her traits *(trait activation*), which may result in positive and/or negative outcomes for him/her and/or for the organization (*outcome activation*). For organizations, job crafting (Wrzesniewski and Dutton, 2001) may take on a negative meaning when employees and leaders with a TNT profile use job crafting to adapt their job and the organization to their personality. That is, during this phase, a TNT leader is likely to want to find a personal niche in the organization or to change his/her job and organization for egocentric reasons (a) in order to enrich him-/herself (dishonesty), (b) in order to have no restrictions when dealing with people who oppose him/her (disagreeableness), and (c) in order to be unhampered by rules, regulations, plans, and goals (carelessness). As an example, narcissistic (i.e., high extraversion and low honesty-humility) CEOs have been found to be able to increase the earning gap between them and the other top managers in their team (O'Reilly et al., 2014).

What can an organization do to prevent TNT leaders from inflicting harm on the organization? First and foremost, surveillance and an ethical culture have been found to be negatively related to delinquent work behaviors (de Vries and Van Gelder, 2015). Top managers' ethical leadership was found to have a “trickle-down” effect through supervisory ethical leadership on employees' OCBs, organizational commitment, and reduced deviance two hierarchical levels down (Mayer et al., 2009; Ruiz et al., 2011). The reverse is also true. Abusive management was found to have a trickle-down effect on employees two hierarchical levels down, such that work group interpersonal deviance was higher in employees when management used an abusive leadership style (Mawritz et al., 2012). Second, activation of the TNT is more likely when TNT behaviors are rewarded. Compared to people high on honesty-humility, people low on honesty-humility were more likely to cheat or to contribute less to a public good when there was no chance of being caught (Hilbig and Zettler, 2015) and when punishment for uncooperative behaviors was unlikely (Hilbig et al., 2012). Additionally, people high on honesty-humility were more likely to be cooperative than people low on honesty-humility when others were cooperative as well (Zettler et al., 2013). Thus, when higher management sets an ethical example, supports virtuous behaviors, and makes sure negative consequences result from counterproductive (TNT) behaviors, it is less likely that TNT—and more likely that virtuous—behaviors are activated.

### Promotion

Promotion is an important outcome for those with a TNT profile, because higher positions are more likely to be accompanied with a higher income and more status, power, and autonomy (*outcome activation*), which are associated with fewer constraints on trait expression (Galinsky et al., 2008). Especially those low on honesty-humility are more likely to use impression management techniques (e.g., ingratiating superiors) in politicized organizations, which may ultimately help them to advance (Wiltshire et al., 2014). The higher the position, the more harm a TNT leader can do to the organization, and thus the more important it is to have adequate promotion selection mechanisms in place.

For promotion the same applies as for selection, but generally more information about the person from within the organization is available during a promotion trajectory, and thus in theory it should be easier for an organization to determine whether the TNT are present or not. However, during this phase, the organization can mistakenly interpret TNT behaviors in terms of leadership attributes, i.e., leader dishonesty in terms of “cunningness,” leader disagreeableness in terms of “toughness,” and leader carelessness in terms of “willingness to delegate.” Furthermore, the organization can mistakenly only rely on supervisory instead of 360° reports. Whereas TNT leaders are less likely to let their supervisors become aware of dishonest, disagreeable, and possibly even careless behaviors, subordinates are more likely to be confronted with such behaviors. Ambition, which is related to career success and a higher income (Ashby and Schoon, 2010; Judge and Kammeyer-Mueller, 2012), is often regarded as a positive attribute, but it may also be indicative of greed, a facet of low honesty-humility (Lerman, 2002; Lee and Ashton, 2004). Arguably, organizations should select on humility instead. Leader humility has been found to improve interpersonal team processes, which, in turn, has been found to result in greater team performance (Owens and Hekman, 2016).

### Attrition

Another possible outcome of the TNT is a person's voluntary or involuntary attrition (*outcome activation*). Meta-analyses and longitudinal studies have shown that job performance is negatively related to turnover (McEvoy and Cascio, 1987; Williams and Livingstone, 1994; Griffeth et al., 2000; Zimmerman and Darnold, 2009), and this relation seems to be true even when turnover is involuntary (Shaw et al., 1998). Consequently, organizations seem to rely to some extent on job performance indicators to discharge dysfunctional personnel. However, as noted above, some TNT employees, such as psychopaths, seem to be found relatively frequently in the boardroom (Babiak et al., 2010), suggesting that not all organization are able to adequately deal with low performing managers. Research suggests that organizations that have a highly develop HR system with high selection rates (Shaw et al., 1998) and performance-contingent rewards (Williams and Livingstone, 1994; Griffeth et al., 2000) have a stronger relation between job performance and turnover, and thus more extensive HR systems may be associated with a reduced chance for TNT employees to turn into TNT boardroom members.

## How bad is TNT leadership?

Are TNT leaders uniformly bad? And how bad are they? In the following section, I'll discuss (a) possible situations in which nightmare traits may have positive consequences and (b) whether “bad is stronger than good” when talking about leader nightmare traits.

According to some authors, Dark Triad traits (psychopathy, narcissism, and Machiavellianism) in leaders may be beneficial in some contexts (Judge et al., 2009; Spain et al., 2016). According to Judge et al. (2009), the strategic and flexible use of people, resources, and influence tactics by Machiavellian leaders may be associated with positive outcomes for themselves and for their followers. For instance, Machiavellianism among US presidents has been found to be positively related to rated performance (Deluga, 2001) and to the number of legislative achievements (Simonton, 1986). When operating in a corrupt environment, it may be impossible to rise through the ranks and be effective as a leader without being tainted by corruption. For instance, in a case study of political leadership in Lebanon, Neal and Tansey (2010) showed that Rafik Hariri could only rebuild Beirut with “effective corrupt leadership.” In some instances, narcissism has also been equated with greatness. When a work-related area is important for their self-esteem, narcissists may be especially strongly motivated to do their best (Harms et al., 2011). Furthermore, narcissists are more likely to favor attention-grabbing, big, and bold actions; actions that may result in large gains or large losses (Chatterjee and Hambrick, 2007). And finally, managers and executives with psychopathic profiles, although less positively rated on performance and management style, were found to be rated more positively than those lower on psychopathy on communication skills and strategic thinking (Babiak et al., 2010).

The findings on the Dark Triad, however, need to be treated with caution, because some of these findings may be indicative of the effects of other trait dimensions than the TNT. That is, some of the positive effects noted above may be due to the positive effects of extraversion or cognitive abilities instead. It may be true that only highly extravert and intelligent TNT leaders are able to make it to the top, being able to adequately “neutralize” the accusations and conflicts that they encounter on the way up. As noted above, the effects of narcissism on leader emergence disappeared once the effects of extraversion were controlled (Grijalva et al., 2015). Similarly, potential positive effects of narcissism on leader effectiveness may disappear when controlled for extraversion. Note that earlier, I argued that extraversion may aggravate the relations between the TNT and outcomes. Some of these negative (fraudulent, self-enhancing, chaotic) effects may be especially apparent when the environment is conducive of such leadership (Padilla et al., 2007) but not when sufficient checks and balances are in place to control for the toxic effects of TNT leadership. When sufficient checks and balances are in place, extraversion may account for most if not all of the leadership effects, which may thus turn out to be positive (Judge et al., 2002) rather than negative.

Because the Dark Triad are most strongly related to (low) honesty-humility, these findings may indicate that in some circumstances leader dishonesty may have positive consequences, although it is questionable whether the results are as positive for the team, organization, or society as they are for the leader him-/herself. With respect to leader disagreeableness, it may be an effective conflict strategy for a powerful leader (Sell et al., 2009), although it is questionable whether the short-term gains associated with leader disagreeableness are not offset by long-term losses, associated with higher levels of task and relationship conflicts (Bono et al., 2002; De Dreu and Weingart, 2003; De Wit et al., 2012). With respect to leader carelessness, one might argue that some leaders might get their work done by delegating responsibilities, especially in “mature” teams (Hersey and Blanchard, 1996). But even such delegation would entail an active instead of a careless or laissez-faire response of the leader, the latter which is generally found to be generally ineffective (Einarsen et al., 2007). Thus, although in some specific contexts (e.g., in corrupt environments, when resolving a conflict in a powerful position, and/or when dealing with a “mature” team), leader dishonesty, disagreeableness, and carelessness may have less negative or even somewhat positive consequences, overall the effects of TNT leadership seem to be mostly negative.

Is “bad stronger than good” when applied to leadership? Baumeister et al. (2001) have argued that bad events have a stronger effect than good events and that this holds across a broad range of psychological phenomena. It is well-documented that ethical, transformational, supportive, and instrumental leadership are positively related to individual and organizational outcomes such as subordinate satisfaction and team or organizational effectiveness (e.g., Judge and Piccolo, 2004; Judge et al., 2004; Burke et al., 2006; Dumdum et al., 2013). But what does this entail for the nightmare traits? Some scholars have compared the effects of constructive leadership styles (e.g., individualized consideration) with destructive leadership styles (e.g., abusive supervision) but did not find support for the “bad leadership is stronger than good leadership” notion (Schyns and Schilling, 2013; Brandebo et al., 2016). However, to conclude, based on these studies, that bad is not stronger than good, may be premature. For a proper investigation of this notion, it is not adequate to compare the effect sizes of constructive and destructive operationalizations of leadership. Instead, one should compare the effects of both constructive and destructive operationalizations of leadership at the negative pole of outcomes with those at the positive pole of outcomes. For instance, one should investigate whether those who have a leader low on constructive leadership (or: high on destructive leadership) suffer more from the negative consequences (when compared to a neutral position) than those who have a leader high on constructive leadership (or: low on destructive leadership) gain from the positive consequences (when compared to a neutral position). That is, good and bad leadership should be treated as a bipolar continuum, in which gains from the “positive” pole are compared to losses from the “negative” pole to find out whether bad is stronger than good.

## Conclusions and discussion

Surprisingly enough, given the similar background, items, and genetic origin of leadership styles and personality traits and given the fact that leadership behaviors are a subset of behaviors referred to in personality models, only relatively few scholars (Kirkpatrick and Locke, 1991; Hogan and Kaiser, 2005; de Vries, 2008, 2012; Judge et al., 2009; Antonakis et al., 2012; Zaccaro, 2012) have called for a closer integration of leadership and personality research. Even though personality perspectives on leadership have been around for some time (e.g., Stogdill, 1948), a unifying perspective is still lacking. Especially when considering the overwhelming number of (dark) leadership styles that have been proposed, an integration of these two perspectives is more than ever needed. In this article, I suggest that an integration of the dark side of leadership with personality can be achieved by considering three so-called nightmare traits, leader dishonesty, leader disagreeableness, and leader carelessness. First of all, I have argued that commonly used leadership styles can be considered contextualized personality traits. Operationalizations of (dark) leadership styles are highly similar to operationalizations of personality, albeit in a contextualized format. Second, I have shown that low levels of three HEXACO traits, honesty-humility, agreeableness, and conscientiousness, underlie the main negative effects of the destructive leadership styles proposed in the literature (e.g., abusive, despotic, authoritarian, laissez-faire, etc. leadership). Third, I have argued that these TNTs, when combined with high extraversion and low emotionality, may have even greater destructive effects (cf. the effects of psychopathic-narcissistic leadership). Fourth, I have introduced the STOA model to account for the process by which the nightmare leadership traits manifest themselves. Fifth and subsequently, I have used the STOA model to delineate the actual effects of TNT leadership in organizations and how to react to them throughout six career phases, i.e., attraction, selection, socialization, production, promotion, and attrition. And finally, I have discussed potential positive effects of the TNT and whether bad leadership is stronger than good leadership.

Although great strides have been made in our understanding of personality and (nightmare) leadership, there are still several research gaps to be filled. First of all, research is warranted which integrates leadership styles—or leadership-contextualized personality—with non-style leadership research, such as research on leader (emotional) intelligence (Cavazotte et al., 2012), leader expertise (Podsakoff et al., 1983), and motivation to lead (Chan and Drasgow, 2001). Whereas cognitive ability has been found to be by-and-large unrelated to personality (Joseph and Newman, 2010), intelligence has been found to be related to general perceptions of leadership (Lord et al., 1986) and to perceptions of transformational leadership (Cavazotte et al., 2012), although ability-based emotional intelligence has not been found to be related to transformational leadership when ratings were derived from different sources (Harms and Cred, 2010). Furthermore, personality—especially extraversion and agreeableness—has been found to be related to the motivation to lead (Chan and Drasgow, 2001). A further integration of leadership-contextualized personality (or leadership styles), competence, motivation, and affect perspectives on leadership is warranted to explain specific leader behaviors and outcomes. Such an integration necessitates large-scale multi-time, multi-methods, multi-raters generalizability studies (Shavelson et al., 1989) to disentangle different sources of variance and to estimate the strength of the relations between leaders' contextualized personality/style, competence, motivation, affect, specific behaviors, and outcomes.

Second, a great number of leadership scales, and especially those that pertain to “dark styles” are problematic because they are highly (negatively) evaluative and pertain to low-base behaviors (e.g., Breevaart and de Vries, 2017). It is known, among others based on studies on low base-rate personality disorders, that answers to items on evaluative scales are more biased than answers to more neutrally formulated items (de Vries et al., 2016a; Ashton et al., 2017). Thus, when creating a contextualized leadership version of the main (HEXACO) personality dimensions, each dimension should preferably be represented by a matched number of positive and negative formulated items, reducing response biases typically observed in answers to leadership questionnaires.

Third, when such a contextualized leadership questionnaire is created, it will be better feasible to disentangle the relative effects of leader dishonesty, leader disagreeableness, and leader carelessness on leader effectiveness and subordinate outcomes. Self-other agreement tends to be higher on personality traits than on leadership styles (de Vries, 2012) and so a first question would be whether this is also true for contextualized leadership scales. Additionally, affect and liking has been found to be strongly related to leader ratings (Brown and Keeping, 2005), so a second question would be whether target variance is increased and relationship variance is decreased in contextualized leadership scales when compared to commonly used leadership instruments (Livi et al., 2008; de Vries, 2010). Furthermore, when using different sources, the next main question would be whether contextualized—and more neutrally formulated—leadership scales are better able to predict important outcomes than existing instruments.

Fourth, with respect to the TNT and the three non-TNT dimensions, an important question would be whether TNT and non-TNT scales interact in the explanation of leadership outcomes. By combining the TNT, non-TNT, and Dark Triad/Tetrad in one analysis, it is also possible to determine whether the effects of the Dark Triad/Tetrad variables are just due to the TNT or to a combination of TNT with non-TNT variables. If the latter is the case, a follow-up question is whether profiles that combine the TNT with high levels of extraversion and low levels of emotionality are more likely to result in worse outcomes for organizations than profiles that combine the TNT with low levels of extraversion and high levels of emotionality. Such an analysis may be problematic, because it would also need to resolve whether checks and balances interact with the outcomes of such profiles. The expectation would be that especially in contexts in which there are insufficient checks and balances, TNT leadership, combined with high extraversion and low emotionality, is especially explosive. Furthermore, investigations of the effects of such profiles over time (i.e., when do the effects of the TNT unfold, and are narcissistic leaders well-liked at first only because of their higher levels of extraversion?) and the differential effects of the TNT on subordinates, colleagues, and supervisors, would greatly help delineating the circumstances in which TNT leadership has the strongest impact.

Fifth, such research would be greatly helped if we could find out what organizations in which industries are more likely to be attractive to TNT applicants to leadership positions. In line with the STOA model, I have argued that organizations that offer greater opportunities for quick advancement, freewheeling, and quick monetary gains, which are slack on goal-setting and planning, which have a lower levels of surveillance, and which see harsh treatment as a sign of leadership, are more likely to be attractive to TNT leaders because such organizations fully allow them to freely express their traits and to gain desirable outcomes from these traits. The HR department in organizations might benefit from a full analysis of each of their career stages in order to find out whether they attract, select, socialize, promote, or (fail to) attrite TNT leaders.

Sixth and finally, more research needs to be carried out to distinguish circumstances in which TNT leadership may play a positive role and whether “bad” leadership is really worse than “good” leadership. As argued above, the latter should be investigated using another design than a design in which the effect sizes of destructive leadership styles are compared to the effect sizes of constructive leadership styles (Schyns and Schilling, 2013; Brandebo et al., 2016). Note, however, that this might be hard to ascertain, because one would have to carefully delineate what a “neutral” leadership effect is and what the objective costs and benefits of destructive and constructive leadership styles are.

There are certain aspects in our current time that seem highly beneficial for TNT leaders in organizations, i.e., in a global world, it is easier to select niches that allow some people to exploit a great number of other people; organizations can grow tremendously practically overnight, and because of the fast pace of change, it is practically impossible to control our most important resource, the people who work in our organizations and the leaders who influence them. Awareness of the leadership traits that make organizations a nightmare to work in, may constitute the first step in preventing an important reason for stress and burnout among employees (Schyns and Schilling, 2013). Distinguishing the three most important traits that seem to underlie the dark side of leadership—leader dishonesty, leader disagreeableness, and leader carelessness—, and getting a grip on the steps that organizations can take to deal with these traits, may go a long way in helping create a more optimal work environment.

## Author contributions

The author confirms being the sole contributor of this work and approved it for publication.

### Conflict of interest statement

A Dutch version of the HEXACO-PI-R has been released for commercial purposes. A percentage of the profit from sales is used by the University to support the research of the author.

## Footnotes

^1^In contrast to research on the stability of personality, studies that have investigated the stability of leadership styles are relatively few. Apart from Harris and Fleishman (1955), the other three referenced articles included two waves of leadership measurement in the context of a longitudinal design, but these articles only used single subordinate reports of leadership.

^2^Self-other agreement on personality is much higher among close friends (*r* = 0.47 in Connelly and Ones, 2010, and *r* = 0.59 in de Vries et al., 2008), family members (0.49 and 0.62), and partners (0.58 and 0.69).

^3^In contrast to most leadership constructs, personality constructs are conceptualized using items that cover both poles of the constructs, suggesting that low levels of a trait (e.g., introversion) are the opposite of high levels of that same trait (e.g., extraversion). Operationalizations of dimensional constructs that include items from both poles have the advantage that they reduce response biases (Ashton et al., 2017). In this manuscript, traits are conceptualized as *density distributions*, i.e., the tendency to act in one way or another in terms of the likelihood/frequency of trait-related behaviors. For example, a dishonest leader may very well often act in an honest way. However, as I will explain in section The STOA Model of TNT Leadership and Table 1, such a leader will be more likely to act in a dishonest way than an honest leader if the trait gets activated in a situation that allows for deception.

^4^This Table is an—for TNT leadership—adapted version of Table 1 in de Vries (2016).
